# 3-*tert*-Butyl-1*H*-isochromene-1-thione

**DOI:** 10.1107/S1600536810019422

**Published:** 2010-05-26

**Authors:** F. Nawaz Khan, P. Manivel, S. Mohana Roopan, Venkatesha R. Hathwar, Mehmet Akkurt

**Affiliations:** a Organic and Medicinal Chemistry Research Laboratory, Organic Chemistry Division, School of Advanced Sciences, VIT University, Vellore 632 014, Tamil Nadu, India; b Solid State and Structural Chemistry Unit, Indian Institute of Science, Bangalore 560 012, Karnataka, India; c Department of Physics, Faculty of Arts and Sciences, Erciyes University, 38039 Kayseri, Turkey

## Abstract

The title compound, C_13_H_14_OS, crystallizes with two independent mol­ecules in the asymmetric unit. The unit cell contains three voids of 197 Å^3^, but the residual electron density (highest peak = 0.24 e Å^−3^ and deepest hole = −0.18 e Å^−3^) in the difference Fourier map suggests no solvent mol­ecule occupies this void. The crystal structure is stabilized by π–π inter­actions between the isocoumarin ring systems, with centroid–centroid distances of 3.6793 (14) and 3.6566 (15) Å.

## Related literature

For the crystal structure and synthesis of isocoumarin and its thio­analogues, see: Hathwar *et al.* (2007*a* ▶,*b* ▶, 2009 ▶); Manivel *et al.* (2008 ▶); Basvanag *et al.* (2009 ▶); Henerson & Hill (1982 ▶).
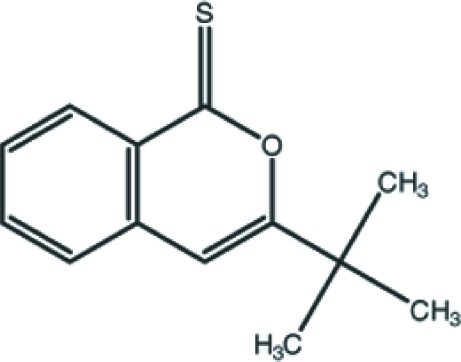


## Experimental

### 

#### Crystal data

C_13_H_14_OS
*M* *_r_* = 218.31
Trigonal, 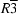

*a* = 43.2799 (16) Å
*c* = 6.9025 (5) Å
*V* = 11197.2 (10) Å^3^
*Z* = 36
Mo *K*α radiation
μ = 0.23 mm^−1^
*T* = 293 K
0.31 × 0.18 × 0.15 mm

#### Data collection

Bruker SMART CCD area-detector diffractometer
Absorption correction: multi-scan (*SADABS*; Sheldrick, 1996 ▶) *T*_min_ = 0.932, *T*_max_ = 0.966
33028 measured reflections
5943 independent reflections
3409 reflections with *I* > 2σ(*I*)
*R*_int_ = 0.047

#### Refinement

*R*[*F* ^2^ > 2σ(*F* ^2^)] = 0.074
*wR*(*F* ^2^) = 0.161
*S* = 1.09
5943 reflections
271 parameters
H-atom parameters constrained
Δρ_max_ = 0.24 e Å^−3^
Δρ_min_ = −0.18 e Å^−3^

### 

Data collection: *SMART* (Bruker, 2004 ▶); cell refinement: *SAINT* (Bruker, 2004 ▶); data reduction: *SAINT*; program(s) used to solve structure: *SIR97* (Altomare *et al.*, 1999 ▶); program(s) used to refine structure: *SHELXL97* (Sheldrick, 2008 ▶); molecular graphics: *ORTEP-3 for Windows* (Farrugia, 1997 ▶); software used to prepare material for publication: *WinGX* (Farrugia, 1999 ▶) and *PLATON* (Spek, 2009 ▶).

## Supplementary Material

Crystal structure: contains datablocks global, I. DOI: 10.1107/S1600536810019422/fj2306sup1.cif

Structure factors: contains datablocks I. DOI: 10.1107/S1600536810019422/fj2306Isup2.hkl

Additional supplementary materials:  crystallographic information; 3D view; checkCIF report

## Figures and Tables

**Fig. 1.**
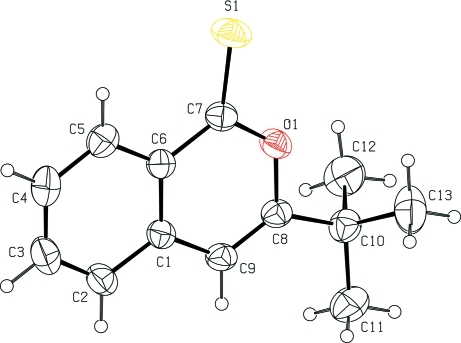
The first molecule in the asymmetric unit with the atom numbering scheme. Displacement ellipsoids for non-H atoms are drawn at the 30% probability level.

**Fig. 2.**
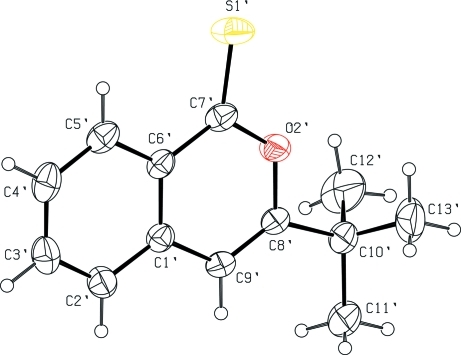
The second molecule in the asymmetric unit with the atom numbering scheme. Displacement ellipsoids for non-H atoms are drawn at the 30% probability level.

**Fig. 3.**
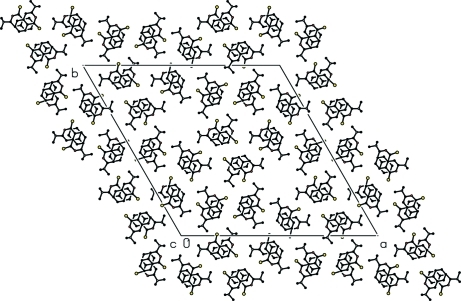
The packing diagram of the title compound down c axis in the unitcell.

## supplementary crystallographic information

### Comment

Isocoumarins are isolated in a great variety of microorganisms, plants, and insects, and have been shown to have considerable biological activity. Isocoumarins and its derivatives are secondary metabolites of a wide variety of microbial plant and insect sources and in synthesis of other medicinal compounds (Manivel *et al.*, 2008, Basvanag *et al.*, 2009). Sulfur containing isocoumarins have been known and a number of substituted thioisocoumarins, (Henerson *et al.*, 1982) have been prepared. Most methods available for the construction of thioisocoumarin nucleus suffer from one or more drawbacks, such as the long reaction time required obtaining a good yield of the desired product or the use of expensive and hazardous reagents and solvents. Herewith, we are reporting the synthesis of 3-*tert*-butyl-1*H*-isochromene-1-thione using Lawessons reagent.

The isocoumarin moieties in the symmetric unit of the title compound, (I), (Fig. 1a and Fig. 1 b), are essentially parallel to each other with a small dihedral angle of 1.20 (7) °.

In the molecular structure of (I), there exist C—H···S and C—H···O intramolecular interactions (Table 1, Fig. 2). In adition, π-π interactions are observed between the isocoumarin ring systems [*Cg*2···*Cg*4(*x*, *y*, *z*) = 3.6793 (14) Å and *Cg*2···*Cg*5(*x*, *y*, 1 + *z*) = 3.6566 (15) Å; where *Cg*2, *Cg*4 and *Cg*5 are the centroids of the C1–C6, O2'/C1'/C6'–C9' and C1'–C6' rings, respectively]. There is no classic hydrogen bonds in the structure.

### Experimental

The 3-*tert*-butyl-1*H*-isochromen-1-one and Lawessons reagent were taken in toluene (1:1 ratio) and refluxed for 1 h. Then the reaction mass was quenched with water, extracted with dichloromethane, washed with water, dried, concentrated and purified by column chromatography to get the titled compound. Single crystals of the title compound were obtained *via *recrystalization from a chloroform solution.

### Refinement

All H-atoms were placed in calculated positions (C—H = 0.93 and 0.96 Å) and were included in the refinement in the riding model approximation, with *U*_iso_(H) set to 1.2 or 1.5*U*_eq_(C). In the crystal structure, there is an 197 Å^3^ void, but the low electron density (0.24 e.Å^-3^) in the difference Fourier map suggests no solvent molecule occupying this void.

### Crystal data

**Table:**

C_13_H_14_OS	*D*_x_ = 1.166 Mg m^−^^3^
*M**_r_* = 218.31	Mo *K*α radiation, λ = 0.71073 Å
Trigonal, *R*3	Cell parameters from 856 reflections
Hall symbol: -R 3	θ = 1.9–24.7°
*a* = 43.2799 (16) Å	µ = 0.23 mm^−^^1^
*c* = 6.9025 (5) Å	*T* = 293 K
*V* = 11197.2 (10) Å^3^	Block, yellow
*Z* = 36	0.31 × 0.18 × 0.15 mm
*F*(000) = 4176	

### Data collection

**Table:**

Bruker SMART CCD area-detector diffractometer	5943 independent reflections
Radiation source: fine-focus sealed tube	3409 reflections with *I* > 2σ(*I*)
graphite	*R*_int_ = 0.047
ω scans	θ_max_ = 28.0°, θ_min_ = 1.6°
Absorption correction: multi-scan (*SADABS*; Sheldrick, 1996)	*h* = −57→57
*T*_min_ = 0.932, *T*_max_ = 0.966	*k* = −56→56
33028 measured reflections	*l* = −9→9

### Refinement

**Table:**

Refinement on *F*^2^	Primary atom site location: structure-invariant direct methods
Least-squares matrix: full	Secondary atom site location: difference Fourier map
*R*[*F*^2^ > 2σ(*F*^2^)] = 0.074	Hydrogen site location: inferred from neighbouring sites
*wR*(*F*^2^) = 0.161	H-atom parameters constrained
*S* = 1.09	*w* = 1/[σ^2^(*F*_o_^2^) + (0.0599*P*)^2^ + 4.5485*P*] where *P* = (*F*_o_^2^ + 2*F*_c_^2^)/3
5943 reflections	(Δ/σ)_max_ = 0.001
271 parameters	Δρ_max_ = 0.24 e Å^−^^3^
0 restraints	Δρ_min_ = −0.18 e Å^−^^3^

### Special details

**Table:**

Geometry. Bond distances, angles etc. have been calculated using the rounded fractional coordinates. All su's are estimated from the variances of the (full) variance-covariance matrix. The cell esds are taken into account in the estimation of distances, angles and torsion angles
Refinement. Refinement on *F*^2^ for ALL reflections except those flagged by the user for potential systematic errors. Weighted *R*-factors wR and all goodnesses of fit *S* are based on *F*^2^, conventional *R*-factors *R* are based on *F*, with *F* set to zero for negative *F*^2^. The observed criterion of *F*^2^ > σ(*F*^2^) is used only for calculating -*R*-factor-obs etc. and is not relevant to the choice of reflections for refinement. *R*-factors based on *F*^2^ are statistically about twice as large as those based on *F*, and *R*-factors based on ALL data will be even larger.

### Fractional atomic coordinates and isotropic or equivalent isotropic displacement parameters (Å^2^)

**Table:**

	*x*	*y*	*z*	*U*_iso_*/*U*_eq_	
S1	0.81419 (2)	−0.00817 (2)	0.78291 (13)	0.0811 (3)	
O1	0.87826 (4)	0.04385 (4)	0.7757 (3)	0.0606 (7)	
C1	0.86386 (7)	0.09888 (6)	0.7809 (3)	0.0502 (8)	
C2	0.85585 (8)	0.12659 (7)	0.7850 (4)	0.0631 (10)	
C3	0.82145 (9)	0.11923 (8)	0.7911 (4)	0.0729 (11)	
C4	0.79363 (8)	0.08436 (9)	0.7932 (4)	0.0720 (11)	
C5	0.80057 (7)	0.05677 (7)	0.7903 (4)	0.0635 (10)	
C6	0.83556 (6)	0.06358 (6)	0.7841 (3)	0.0492 (8)	
C7	0.84331 (7)	0.03469 (6)	0.7812 (4)	0.0548 (9)	
C8	0.90635 (6)	0.07871 (6)	0.7703 (3)	0.0516 (8)	
C9	0.89948 (7)	0.10530 (6)	0.7739 (3)	0.0535 (8)	
C10	0.94129 (7)	0.07913 (7)	0.7620 (4)	0.0639 (10)	
C11	0.97229 (7)	0.11727 (8)	0.7400 (5)	0.0878 (13)	
C12	0.94624 (9)	0.06357 (9)	0.9523 (5)	0.0979 (16)	
C13	0.94106 (9)	0.05714 (9)	0.5875 (5)	0.1021 (16)	
S1'	0.77849 (2)	0.11691 (2)	0.28420 (12)	0.0772 (3)	
O2'	0.76501 (4)	0.05251 (4)	0.2971 (3)	0.0574 (6)	
C1'	0.83413 (6)	0.06544 (6)	0.2874 (3)	0.0465 (8)	
C2'	0.86926 (6)	0.07253 (7)	0.2822 (4)	0.0574 (9)	
C3'	0.89714 (7)	0.10670 (8)	0.2764 (4)	0.0649 (10)	
C4'	0.89068 (7)	0.13502 (7)	0.2747 (4)	0.0660 (10)	
C5'	0.85652 (7)	0.12899 (6)	0.2800 (3)	0.0579 (9)	
C6'	0.82767 (6)	0.09412 (6)	0.2863 (3)	0.0459 (8)	
C7'	0.79136 (6)	0.08729 (6)	0.2900 (3)	0.0520 (8)	
C8'	0.77110 (6)	0.02397 (6)	0.2971 (4)	0.0521 (8)	
C9'	0.80418 (6)	0.02988 (6)	0.2925 (3)	0.0524 (9)	
C10'	0.73624 (7)	−0.01086 (6)	0.3019 (5)	0.0680 (10)	
C11'	0.74338 (8)	−0.04203 (7)	0.2923 (5)	0.0903 (13)	
C12'	0.71642 (9)	−0.01317 (8)	0.4895 (6)	0.1127 (16)	
C13'	0.71430 (8)	−0.01215 (8)	0.1256 (6)	0.1090 (16)	
H2	0.87430	0.15020	0.78360	0.0760*	
H3	0.81660	0.13790	0.79390	0.0880*	
H4	0.77020	0.07960	0.79660	0.0870*	
H5	0.78180	0.03330	0.79240	0.0760*	
H9	0.91830	0.12870	0.77170	0.0640*	
H11A	0.96880	0.12780	0.62590	0.1320*	
H11B	0.99420	0.11690	0.72860	0.1320*	
H11C	0.97330	0.13100	0.85160	0.1320*	
H12A	0.94830	0.07890	1.05820	0.1460*	
H12B	0.96750	0.06190	0.94440	0.1460*	
H12C	0.92600	0.04030	0.97290	0.1460*	
H13A	0.92160	0.03310	0.59980	0.1540*	
H13B	0.96320	0.05700	0.58270	0.1540*	
H13C	0.93820	0.06750	0.47070	0.1540*	
H2'	0.87380	0.05370	0.28280	0.0690*	
H3'	0.92050	0.11100	0.27370	0.0780*	
H4'	0.90970	0.15830	0.26980	0.0790*	
H5'	0.85250	0.14820	0.27940	0.0700*	
H9'	0.80810	0.01060	0.29270	0.0630*	
H11D	0.75560	−0.04070	0.17380	0.1360*	
H11E	0.72110	−0.06410	0.29670	0.1360*	
H11F	0.75790	−0.04080	0.40050	0.1360*	
H12D	0.72880	−0.01600	0.59730	0.1690*	
H12E	0.69270	−0.03330	0.48330	0.1690*	
H12F	0.71530	0.00830	0.50580	0.1690*	
H13D	0.71030	0.00780	0.12930	0.1630*	
H13E	0.69180	−0.03400	0.12730	0.1630*	
H13F	0.72700	−0.01110	0.00950	0.1630*	

### Atomic displacement parameters (Å^2^)

**Table:**

	*U*^11^	*U*^22^	*U*^33^	*U*^12^	*U*^13^	*U*^23^
S1	0.0696 (5)	0.0415 (4)	0.1141 (7)	0.0142 (3)	−0.0067 (4)	−0.0026 (4)
O1	0.0547 (11)	0.0442 (10)	0.0807 (13)	0.0231 (8)	−0.0041 (9)	−0.0046 (8)
C1	0.0629 (16)	0.0480 (14)	0.0398 (14)	0.0278 (13)	0.0024 (11)	−0.0030 (11)
C2	0.082 (2)	0.0539 (16)	0.0593 (17)	0.0383 (15)	0.0031 (14)	−0.0037 (13)
C3	0.100 (2)	0.079 (2)	0.0639 (19)	0.063 (2)	0.0030 (16)	−0.0045 (15)
C4	0.0701 (19)	0.097 (2)	0.0658 (19)	0.0544 (19)	0.0005 (15)	−0.0057 (17)
C5	0.0550 (16)	0.0694 (18)	0.0613 (17)	0.0275 (14)	0.0006 (13)	−0.0017 (14)
C6	0.0540 (14)	0.0494 (14)	0.0420 (14)	0.0243 (12)	−0.0004 (11)	−0.0034 (11)
C7	0.0536 (15)	0.0482 (14)	0.0561 (16)	0.0206 (12)	−0.0047 (12)	−0.0036 (12)
C8	0.0493 (14)	0.0467 (14)	0.0497 (15)	0.0171 (12)	−0.0020 (11)	−0.0034 (11)
C9	0.0575 (15)	0.0429 (13)	0.0497 (15)	0.0173 (12)	0.0015 (12)	−0.0028 (11)
C10	0.0535 (16)	0.0663 (17)	0.0679 (19)	0.0270 (14)	−0.0014 (13)	−0.0039 (14)
C11	0.0572 (18)	0.082 (2)	0.107 (3)	0.0220 (16)	0.0055 (17)	0.0042 (18)
C12	0.080 (2)	0.115 (3)	0.111 (3)	0.058 (2)	0.0003 (19)	0.028 (2)
C13	0.081 (2)	0.117 (3)	0.121 (3)	0.059 (2)	−0.004 (2)	−0.037 (2)
S1'	0.0796 (5)	0.0522 (4)	0.1108 (7)	0.0413 (4)	0.0313 (4)	0.0127 (4)
O2'	0.0444 (9)	0.0391 (9)	0.0862 (13)	0.0191 (8)	0.0055 (8)	−0.0002 (8)
C1'	0.0473 (13)	0.0459 (13)	0.0432 (14)	0.0209 (11)	0.0008 (11)	−0.0034 (10)
C2'	0.0506 (15)	0.0635 (16)	0.0586 (17)	0.0288 (13)	−0.0024 (12)	−0.0038 (13)
C3'	0.0444 (15)	0.077 (2)	0.0610 (18)	0.0212 (14)	−0.0013 (12)	−0.0015 (14)
C4'	0.0506 (16)	0.0582 (17)	0.0604 (18)	0.0055 (13)	0.0034 (13)	−0.0005 (13)
C5'	0.0616 (17)	0.0445 (14)	0.0561 (16)	0.0178 (12)	0.0086 (13)	−0.0021 (12)
C6'	0.0447 (13)	0.0410 (12)	0.0430 (14)	0.0147 (11)	0.0056 (10)	−0.0042 (10)
C7'	0.0559 (15)	0.0414 (13)	0.0536 (16)	0.0205 (12)	0.0112 (12)	−0.0004 (11)
C8'	0.0507 (14)	0.0364 (12)	0.0675 (17)	0.0206 (11)	−0.0047 (12)	−0.0061 (11)
C9'	0.0539 (15)	0.0396 (13)	0.0665 (17)	0.0255 (12)	−0.0043 (12)	−0.0069 (11)
C10'	0.0500 (15)	0.0382 (14)	0.105 (2)	0.0140 (12)	−0.0103 (16)	−0.0065 (14)
C11'	0.0727 (19)	0.0383 (15)	0.147 (3)	0.0181 (14)	−0.023 (2)	−0.0098 (17)
C12'	0.078 (2)	0.061 (2)	0.158 (4)	0.0039 (17)	0.041 (2)	0.006 (2)
C13'	0.072 (2)	0.063 (2)	0.168 (4)	0.0157 (17)	−0.047 (2)	−0.012 (2)

### Geometric parameters (Å, °)

**Table:**

S1—C7	1.641 (2)	C13—H13A	0.9600
S1'—C7'	1.634 (3)	C13—H13B	0.9600
O1—C8	1.386 (3)	C13—H13C	0.9600
O1—C7	1.359 (4)	C1'—C2'	1.393 (4)
O2'—C8'	1.386 (3)	C1'—C6'	1.402 (4)
O2'—C7'	1.361 (3)	C1'—C9'	1.434 (3)
C1—C6	1.401 (3)	C2'—C3'	1.364 (4)
C1—C2	1.405 (4)	C3'—C4'	1.387 (4)
C1—C9	1.424 (5)	C4'—C5'	1.367 (5)
C2—C3	1.359 (6)	C5'—C6'	1.398 (3)
C3—C4	1.382 (5)	C6'—C7'	1.447 (4)
C4—C5	1.369 (5)	C8'—C9'	1.323 (4)
C5—C6	1.391 (4)	C8'—C10'	1.509 (4)
C6—C7	1.448 (4)	C10'—C11'	1.529 (4)
C8—C9	1.325 (4)	C10'—C12'	1.529 (5)
C8—C10	1.504 (4)	C10'—C13'	1.527 (5)
C10—C11	1.528 (4)	C2'—H2'	0.9300
C10—C12	1.539 (5)	C3'—H3'	0.9300
C10—C13	1.532 (4)	C4'—H4'	0.9300
C2—H2	0.9300	C5'—H5'	0.9300
C3—H3	0.9300	C9'—H9'	0.9300
C4—H4	0.9300	C11'—H11D	0.9600
C5—H5	0.9300	C11'—H11E	0.9600
C9—H9	0.9300	C11'—H11F	0.9600
C11—H11A	0.9600	C12'—H12D	0.9600
C11—H11B	0.9600	C12'—H12E	0.9600
C11—H11C	0.9600	C12'—H12F	0.9600
C12—H12C	0.9600	C13'—H13D	0.9600
C12—H12A	0.9600	C13'—H13E	0.9600
C12—H12B	0.9600	C13'—H13F	0.9600
			
S1'···C5'^i^	3.681 (2)	H9···H11A	2.4200
S1···H3'^ii^	3.0200	H9···H11C	2.4000
S1···H11C^iii^	3.0800	H9'···C11'	2.5800
S1···H5	2.7800	H9'···H2'	2.5000
S1'···H11E^iv^	3.1800	H9'···H11D	2.3900
S1'···H5'	2.7900	H9'···H11F	2.3200
S1'···H5'^i^	3.0100	H11A···H9	2.4200
S1'···H2^v^	3.2000	H11A···H13C	2.5000
O1···H13A	2.4700	H11A···C9	2.8400
O1···H12C	2.5400	H11B···H12B	2.5400
O2'···H12F	2.5000	H11B···H13B	2.4600
O2'···H13D	2.4700	H11C···H9	2.4000
C1'···C6	3.431 (3)	H11C···C9	2.8600
C1'···C6^vi^	3.476 (3)	H11C···H12A	2.4200
C2···C5'	3.487 (3)	H11C···S1^ix^	3.0800
C2···C5'^vii^	3.418 (3)	H11D···H13F	2.4600
C3'···C9^vi^	3.471 (3)	H11D···C9'	2.8300
C3'···C9	3.437 (3)	H11D···H9'	2.3900
C4···C7'^vii^	3.435 (3)	H11E···H12E	2.5700
C4···C7'	3.479 (3)	H11E···H13E	2.5100
C5'···S1'^i^	3.681 (2)	H11E···S1'^x^	3.1800
C5'···C2	3.487 (3)	H11F···C9'	2.7900
C5'···C2^vi^	3.418 (3)	H11F···H12D	2.4400
C6···C1'^vii^	3.476 (3)	H11F···H9'	2.3200
C6···C1'	3.431 (3)	H12A···H11C	2.4200
C7'···C4^vi^	3.435 (3)	H12B···H13B	2.5000
C7'···C4	3.479 (3)	H12B···H11B	2.5400
C9···C3'	3.437 (3)	H12C···H13A	2.5900
C9···C3'^vii^	3.471 (3)	H12C···O1	2.5400
C9···H11A	2.8400	H12D···H11F	2.4400
C9···H11C	2.8600	H12D···H5	2.5900
C9'···H11F	2.7900	H12E···H13E	2.4600
C9'···H11D	2.8300	H12E···H11E	2.5700
C11···H9	2.6300	H12F···O2'	2.5000
C11'···H9'	2.5800	H12F···H13E^iv^	2.5900
H2···H9	2.5000	H13A···H12C	2.5900
H2···S1'^v^	3.2000	H13A···O1	2.4700
H2'···H9'	2.5000	H13B···H12B	2.5000
H3'···S1^viii^	3.0200	H13B···H11B	2.4600
H5···H12D	2.5900	H13C···H11A	2.5000
H5···S1	2.7800	H13D···O2'	2.4700
H5'···S1'	2.7900	H13E···H11E	2.5100
H5'···S1'^i^	3.0100	H13E···H12E	2.4600
H9···C11	2.6300	H13E···H12F^x^	2.5900
H9···H2	2.5000	H13F···H11D	2.4600
			
C7—O1—C8	124.1 (2)	H13A—C13—H13B	109.00
C7'—O2'—C8'	123.9 (2)	C2'—C1'—C6'	118.9 (2)
C2—C1—C6	118.4 (3)	C2'—C1'—C9'	122.6 (2)
C2—C1—C9	122.6 (2)	C6'—C1'—C9'	118.5 (3)
C6—C1—C9	119.0 (2)	C1'—C2'—C3'	121.1 (3)
C1—C2—C3	120.6 (3)	C2'—C3'—C4'	119.9 (3)
C2—C3—C4	120.7 (3)	C3'—C4'—C5'	120.5 (3)
C3—C4—C5	120.1 (4)	C4'—C5'—C6'	120.3 (3)
C4—C5—C6	120.4 (3)	C1'—C6'—C5'	119.3 (3)
C1—C6—C7	119.2 (3)	C1'—C6'—C7'	119.7 (2)
C5—C6—C7	121.0 (2)	C5'—C6'—C7'	120.9 (2)
C1—C6—C5	119.8 (2)	S1'—C7'—O2'	116.3 (2)
S1—C7—C6	126.7 (2)	S1'—C7'—C6'	126.93 (18)
O1—C7—C6	117.0 (2)	O2'—C7'—C6'	116.8 (2)
S1—C7—O1	116.3 (2)	O2'—C8'—C9'	119.8 (2)
O1—C8—C10	110.1 (2)	O2'—C8'—C10'	110.5 (2)
C9—C8—C10	130.6 (2)	C9'—C8'—C10'	129.7 (2)
O1—C8—C9	119.3 (3)	C1'—C9'—C8'	121.2 (2)
C1—C9—C8	121.5 (2)	C8'—C10'—C11'	109.8 (3)
C8—C10—C12	108.7 (3)	C8'—C10'—C12'	109.4 (2)
C8—C10—C13	109.2 (3)	C8'—C10'—C13'	108.2 (2)
C8—C10—C11	110.6 (2)	C11'—C10'—C12'	109.5 (3)
C11—C10—C13	108.8 (3)	C11'—C10'—C13'	109.2 (3)
C12—C10—C13	111.0 (3)	C12'—C10'—C13'	110.8 (3)
C11—C10—C12	108.6 (3)	C1'—C2'—H2'	120.00
C3—C2—H2	120.00	C3'—C2'—H2'	119.00
C1—C2—H2	120.00	C2'—C3'—H3'	120.00
C2—C3—H3	120.00	C4'—C3'—H3'	120.00
C4—C3—H3	120.00	C3'—C4'—H4'	120.00
C3—C4—H4	120.00	C5'—C4'—H4'	120.00
C5—C4—H4	120.00	C4'—C5'—H5'	120.00
C6—C5—H5	120.00	C6'—C5'—H5'	120.00
C4—C5—H5	120.00	C1'—C9'—H9'	119.00
C1—C9—H9	119.00	C8'—C9'—H9'	119.00
C8—C9—H9	119.00	C10'—C11'—H11D	109.00
C10—C11—H11B	109.00	C10'—C11'—H11E	109.00
C10—C11—H11C	109.00	C10'—C11'—H11F	109.00
C10—C11—H11A	109.00	H11D—C11'—H11E	110.00
H11A—C11—H11C	110.00	H11D—C11'—H11F	109.00
H11B—C11—H11C	110.00	H11E—C11'—H11F	110.00
H11A—C11—H11B	109.00	C10'—C12'—H12D	110.00
C10—C12—H12A	109.00	C10'—C12'—H12E	109.00
C10—C12—H12B	109.00	C10'—C12'—H12F	110.00
H12A—C12—H12B	109.00	H12D—C12'—H12E	109.00
H12A—C12—H12C	109.00	H12D—C12'—H12F	110.00
C10—C12—H12C	109.00	H12E—C12'—H12F	110.00
H12B—C12—H12C	110.00	C10'—C13'—H13D	109.00
C10—C13—H13B	110.00	C10'—C13'—H13E	109.00
C10—C13—H13C	109.00	C10'—C13'—H13F	109.00
C10—C13—H13A	109.00	H13D—C13'—H13E	110.00
H13A—C13—H13C	109.00	H13D—C13'—H13F	110.00
H13B—C13—H13C	110.00	H13E—C13'—H13F	109.00
			
C8—O1—C7—S1	178.89 (18)	C10—C8—C9—C1	179.7 (2)
C8—O1—C7—C6	−0.9 (4)	O1—C8—C10—C11	175.6 (2)
C7—O1—C8—C9	1.2 (3)	O1—C8—C10—C12	−65.3 (3)
C7—O1—C8—C10	−179.1 (2)	O1—C8—C10—C13	55.9 (3)
C8'—O2'—C7'—S1'	178.1 (2)	C6'—C1'—C2'—C3'	0.1 (4)
C8'—O2'—C7'—C6'	−1.3 (3)	C9'—C1'—C2'—C3'	179.7 (2)
C7'—O2'—C8'—C9'	0.9 (4)	C2'—C1'—C6'—C5'	0.0 (3)
C7'—O2'—C8'—C10'	−178.9 (2)	C2'—C1'—C6'—C7'	179.5 (2)
C9—C1—C2—C3	179.8 (2)	C9'—C1'—C6'—C5'	−179.56 (19)
C2—C1—C6—C5	0.3 (3)	C9'—C1'—C6'—C7'	−0.1 (3)
C6—C1—C2—C3	−0.3 (4)	C2'—C1'—C9'—C8'	−179.9 (2)
C6—C1—C9—C8	0.0 (3)	C6'—C1'—C9'—C8'	−0.4 (3)
C9—C1—C6—C5	−179.8 (2)	C1'—C2'—C3'—C4'	−0.3 (4)
C9—C1—C6—C7	0.3 (3)	C2'—C3'—C4'—C5'	0.4 (4)
C2—C1—C6—C7	−179.6 (2)	C3'—C4'—C5'—C6'	−0.3 (4)
C2—C1—C9—C8	180.0 (2)	C4'—C5'—C6'—C1'	0.1 (3)
C1—C2—C3—C4	−0.1 (4)	C4'—C5'—C6'—C7'	−179.4 (2)
C2—C3—C4—C5	0.4 (4)	C1'—C6'—C7'—S1'	−178.42 (17)
C3—C4—C5—C6	−0.4 (4)	C1'—C6'—C7'—O2'	0.9 (3)
C4—C5—C6—C7	179.9 (2)	C5'—C6'—C7'—S1'	1.1 (3)
C4—C5—C6—C1	0.0 (4)	C5'—C6'—C7'—O2'	−179.7 (2)
C1—C6—C7—O1	0.1 (3)	O2'—C8'—C9'—C1'	0.0 (4)
C5—C6—C7—S1	0.5 (4)	C10'—C8'—C9'—C1'	179.7 (3)
C5—C6—C7—O1	−179.8 (2)	O2'—C8'—C10'—C11'	177.4 (2)
C1—C6—C7—S1	−179.63 (19)	O2'—C8'—C10'—C12'	−62.5 (3)
O1—C8—C9—C1	−0.8 (3)	O2'—C8'—C10'—C13'	58.3 (3)
C9—C8—C10—C11	−4.8 (4)	C9'—C8'—C10'—C11'	−2.4 (4)
C9—C8—C10—C12	114.3 (3)	C9'—C8'—C10'—C12'	117.8 (3)
C9—C8—C10—C13	−124.5 (3)	C9'—C8'—C10'—C13'	−121.5 (3)

Symmetry codes: (i) −*x*+5/3, −*y*+1/3, −*z*+1/3; (ii) *x*−*y*, *x*−1, −*z*+1; (iii) *x*−*y*, *x*−1, −*z*+2; (iv) −*y*+2/3, *x*−*y*−2/3, *z*+1/3; (v) −*x*+5/3, −*y*+1/3, −*z*+4/3; (vi) *x*, *y*, *z*−1; (vii) *x*, *y*, *z*+1; (viii) *y*+1, −*x*+*y*+1, −*z*+1; (ix) *y*+1, −*x*+*y*+1, −*z*+2; (x) −*x*+*y*+4/3, −*x*+2/3, *z*−1/3.

### Hydrogen-bond geometry (Å, °)

**Table:**

*D*—H···*A*	*D*—H	H···*A*	*D*···*A*	*D*—H···*A*
C5—H5···S1	0.93	2.78	3.148 (3)	105
C5'—H5'···S1'	0.93	2.79	3.149 (3)	104
C12—H12C···O1	0.96	2.54	2.891 (5)	102
C12'—H12F···O2'	0.96	2.50	2.879 (4)	103
C13—H13A···O1	0.96	2.47	2.800 (5)	100
C13'—H13D···O2'	0.96	2.47	2.812 (4)	101
